# Screening for individuals with postpartum depression by an attenuated and delayed niacin skin flushing response: a case control study

**DOI:** 10.1186/s12884-025-08040-x

**Published:** 2025-09-24

**Authors:** Dandan Wang, Yan Gao, Zhendong Xu, Fuyi Shen, Xiaowen Hu, Xuhan Yang, Qian Wang, Juan Zhang, Jie Jiang, Yan Zhuang, Lize Xiong, Jun Li, Chunling Wan, Zhiqiang Liu

**Affiliations:** 1 Bio-X Institutes, Key Laboratory for the Genetics of Developmental and Neuropsychiatric Disorders, Ministry of Education, Shanghai Jiao Tong University, 1954 Huashan Road, Shanghai, 200030 China; 2 Department of Anesthesiology, Shanghai First Maternity and Infant Hospital, Tongji University School of Medicine, Shanghai, 200040 China; 3 Shanghai Center for Women and Children’s Health, Shanghai, China; 4 Department of Obstetrics and Gynecology, Shanghai General Hospital, Shanghai, China; 5 Department of Anesthesiology and Perioperative medicine, Shanghai Fourth People’s Hospital, School of Medicine, Tongji University, Shanghai, China; 6 Department of Psychology, Changning Maternity and Infant Health Hospital, East China Normal University, Shanghai, China; 7 Department of Anesthesiology, Obstetrics and Gynecology Hospital of Fudan University, 200082 Shanghai, China

**Keywords:** Postpartum depression, Niacin skin flushing, Natural population, Clinical auxiliary screening methods, Disease prognosis

## Abstract

**Background:**

Postpartum depression (PPD) affects maternal mental health extensively and is challenged by the lack of objective diagnostic methods. This study aimed to explore the characteristics of niacin skin flush response, a diagnostic marker for depression, in individuals with PPD and to determine its clinical potential as an adjunctive screening marker.

**Methods:**

A total of 1417 parturients was recruited in this study. Edinburgh Postnatal Depression Scale was used to screen for depression with a cut-off score ≥ 13. The Patient Health Questionnaire-9 scale was used to test the consistency of scale screening. The Chi-square test was used to compare the screening results of the two scales, and the reliability and validity of the two scales were discussed. Mann Whitney *U* test was used to analyze the differences in niacin-flushing between PPD and healthy controls (HC), and a ten-fold cross-validation with logistic regression was used to verify the potential of niacin-flushing to distinguish between PPD and HC. A screening model for women with PPD was established by bivariate truncation method.

**Results:**

The results of the two depression screening scales were 12.85% inconsistent, indicating the nonobjectivity of the scale screening. Compared with the healthy women, women with PPD exhibited significantly attenuated and delayed niacin-induced flushing response. An innovative and user-friendly postpartum depression screening model was established according to the features, by which we could identify women with PPD from healthy women with a sensitivity of 53.09% (95% CI: 42.22% − 63.95%) and a specificity of 73.28% (95% CI: 70.90% − 75.65%).

**Conclusions:**

This study identified the characteristics of impaired niacin response in women with PPD, and established a potential screening standard for PPD, which is highly objective and operable. The niacin-blunted subgroup of PPD may imply a common biological basis, which provokes new thoughts in elucidating the pathological mechanisms of postpartum depression.

**Supplementary Information:**

The online version contains supplementary material available at 10.1186/s12884-025-08040-x.

## Introduction

Postpartum depression (PPD) is the most common mental disorder after childbirth [1]. It is a depressive state that occurs during a specific period. The predisposing factors of PPD are highly heterogeneous, including genetic susceptibility, psychiatric history, drug abuse, lack of social support, poor family relationships, physiological factors, and so on [2]. The consequences of PPD are very serious. In extreme cases, it can lead to self-harm and suicide of the parturient, or even extended suicide [3, 4]. In addition to the mothers themselves, studies have found that newborns of patients with PPD are more likely to suffer from developmental disorders than those of healthy mothers [5].

Currently, the screening of PPD relies heavily on the depression screening scale, including the Edinburgh Postnatal Depression Scale (EPDS), the Patient Health Questionnaire-9 (PHQ-9) scale, and so on [6]. PPD affects approximately 13%−19% of women in the puerperium [7]. In different studies, the prevalence of PPD varies with different measurement tools and diagnostic criteria. Not only that, but there is no consensus on the PPD screening time window [8]. Identifying PPD patients by objective and standard method has become a key issue that plagues the clinical diagnosis and treatment of PPD. Mining PPD objective auxiliary diagnostic markers will become an effective way to solve the issue.

From the 1960 s to the present, the niacin skin flushing response has demonstrated its potential as a clinical marker of psychiatric disorders, including major depressive disorder (MDD), bipolar disorder (BPD) and schizophrenia (SZ) [9–11]. The molecular mechanism of skin flushing caused by niacin stimulation has now been clarified. During the niacin-flushing process, niacin binds to G protein-coupled receptors 109 A (GPR109A) in epidermal cells to activate phospholipase A2 (PLA2) [12, 13]. This mediates the dissociation of arachidonic acid (AA) on the cell membrane into a free state, then the free AA is converted into prostaglandin D2 (PGD2) and prostaglandin E2 (PGE2) under the action of cyclooxygenase enzymes [14]. Prostaglandins can dilate skin capillaries and accelerate blood flow [15]. Eventually, a flushing reaction occurs in the skin irritated by niacin. Damage to any link of this niacin-AA-prostaglandins pathway will result in attenuation of the skin-flushing, which is the phenotype of niacin bluntness. Studies have found that varying degrees of niacin bluntness is widespread in patients with MDD [10, 16]. The screening criteria established based on the niacin bluntness phenotype can screen MDD patients from healthy controls with a sensitivity of 53% and a specificity of 83%, which suggested a promising prospect of clinical applicable potential of the niacin skin flushing response as an objective auxiliary diagnostic biomarker of MDD [10]. PPD is a depressive state that occurs in a specific period. The screening effect of niacin bluntness on patients with MDD provides a new perspective for the exploration of objective diagnostic markers for PPD.

In this study, a natural population study cohort of women at 6 weeks after childbirth was recruited. By exploring the characteristics of niacin skin flushing response in this population, we aimed to clarify the degree of niacin bluntness in patients with PPD and try to establish a clinical auxiliary screening marker for patients with PPD based on the niacin-induced skin flushing, which would provoke new thoughts in elucidating the clinical management and pathological mechanisms of PPD.

## Methods and materials

### Participants

Participants in this study were all recruited in Shanghai First Maternity and Infant Hospital, women 6 weeks after delivery meet the inclusion criteria for this study. Exclusion criteria for all individuals included: (a) other neurological diseases, such as intellectual disabilities, epilepsy, and stroke; (b) history of significant head injury or brain tumors; (c) substance dependence; (d) use of nonsteroidal or steroidal anti-inflammatory drugs within the past week; and (e) serious skin diseases or immune system diseases, such as lupus and asthma. All enrolled participants have no history of mental disorders. All participants signed informed consent forms. The study was approved by the ethics committee of Shanghai First Maternity and Infant Hospital (Ethics acceptance number: KS20302).

### Depression screening

The Chinese version of EPDS scale was employed as the primary screening tool for PPD [17, 18]. In line with established guidelines, a score of ≥ 13 on the EPDS was defined as a positive screen for PPD. An EPDS score of less than 13 was defined an emotional health control (HC). PHQ-9 scale was used to test the reliability and validity of EPDS. A PHQ-9 score greater than 4 indicated depression, 5–9 which were mild depression, and 10 or more were moderate to severe depression.

### Niacin test

Home-made modified niacin patches were prepared for the niacin test. The patches presented a sandwich-like structure, with foam, filter paper and skin-friendly gel from top to bottom. The corresponding positions of the foam layer and the skin-friendly gel layer were evenly distributed with 6 circular holes with a diameter of 1 cm. The filter paper at the position of the round holes severed as the carrier of the niacin solution. This modified patch ensures an adequate amount of solution contacting the skin, because the excess solution would drench the surrounding filter paper which was coated with gels to avoid access to the skin. Equal volumes of aqueous methyl nicotinate (AMN, C_7_H_7_NO_2_, 99%, Sigma-Aldrich) with 6 concentrations (60 mM, 20 mM, 6.67 mM, 2.22 mM, 0.74 mM, and 0.25 mM) each were added into the holes of the patch respectively for topical contact to the subject’s forearm for 1 min, and then the patch was removed. After that, the subject’s forearm was photographed at every 10 s within the whole span of 10 min, and eventually, 60 photos were taken for each individual.

Niacin flushing scores of each individual were obtained by assessing the size of niacin-induced erythema by the 4-point scale established by Ward et al.: 0 = no erythema, 1 = incomplete erythema, 2 = complete erythema within the defined area of the patch, and 3 = erythema beyond the defined area of the patch [19]. The flush responses were evaluated by at least two research assistants independently using Adobe Photoshop (version CS5, Adobe). All research assistants performing erythema scoring were fully blinded to participants’ group status The intraclass correlation coefficient of the scores rated by the two assistants was above 0.90, indicating a good inter-rater reliability. A third assistant was introduced for those discrepancies. Calculate the total flushing score on 60 photos of each subject as the degree of niacin-flushing of the subject. The onset time of erythema development in each subject at various concentrations represents the individual’s response to niacin stimulation.

### Data analysis

Descriptive characteristics of all included participants are provided, and variables are presented as mean (SD, or SEM) values for continuous variables, and proportions and percentages are presented for categorical variables. In the group comparisons of continuous variables, the Kolmogorov-Smirnov (K-S) test was used to assess the normality of the data. The magnitude of differences between groups was expressed as Fold Change (FC), where FC > 1 indicates that the level of nicotinic acid response in the PPD group was higher than that in the healthy control group, while FC < 1 indicates a lower level. For non-normally distributed data, the Mann-Whitney *U* test was applied, and for normally distributed data, the Student’s t-test was used. To account for multiple testing and control the potential increase in Type I errors, we applied the Benjamini-Hochberg (BH) procedure to adjust p-values. The BH method controls the False Discovery Rate (FDR), which is the expected proportion of false positives among all significant results. The adjusted p-values were then used to determine statistical significance at a threshold of α = 0.05. This correction ensures that the overall FDR is controlled at 5%, reducing the likelihood of false positive findings while maintaining statistical power. For comparing the distribution differences of categorical variables between groups, the Chi-square test was used.

To assess the potential of niacin-flushing phenotype in distinguishing between PPD and HC groups, a ten-fold cross-validation approach combined with binary logistic regression was employed. For each fold, the model’s performance was evaluated on the test set using the Receiver Operating Characteristic (ROC) curve and the Area Under the Curve (AUC) with 95% confidence intervals (CIs) computed using the DeLong method. All statistical analyses as indicated were conducted with the SPSS software (Version 22, IBM Corp.) and R Studio (version R4.1.2), and *p* < 0.05 was considered statistically significant.

## Results

### Demographic characteristics

In this study, 1417 postpartum women were recruited without biases, including 1336 healthy women, and 81 PPD women. All of these women had complete demographic data and niacin-induced skin flushing data. Table 1 summarizes the demographic characteristics of each group. There was no significant difference between PPD and HC groups in age, education, BMI, increased weight, obstetric and pediatric factors. Notably, the feeding methods of infants in PPD group was significantly different from the HC group. Specifically, more breastfeeding women stayed in healthy status (93.41%) compared to breastfeeding women had PPD (86.42%) (*p* < 0.05, Chi-square test).

**Table 1:** Demographic characteristics

Characteristics	HC	PPD
Total number, n	1336	81
EPDS^a^	4.623 (2.956)	15.346 (2.807)^***^
PHQ-9^a^	2.685 (2.589)	10.370 (4.692) ^***^
Age, y, mean (SD)^a^	30.489 (3.640)	30.395 (3.073)
Education level, mean (SD)^a^	3.852 (0.453)	3.802 (0.485)
Pregestational BMI, kg/m^2^, mean (SD)^a^	21.452 (3.476)	20.948 (3.040)
Prenatal BMI, kg/m^2^, mean (SD)^a^	26.460 (4.127)	26.219 (3.824)
Postpartum BMI, kg/m^2^, mean (SD)^a^	23.056 (3.458)	22.946 (3.452)
Increased weight during pregnancy, kg, mean (SD)^a^	13.013 (6.997)	13.863 (7.205)
Complications during pregnancy/puerperium		
Pregnancy thyroid disease, n (%)^b^	41 (3.07%)	3 (3.70%)
Gestational diabetes mellitus, n (%)^b^	123 (9.21%)	10 (12.35%)
Gestational hypertension, n (%)^b^	54 (4.04%)	2 (2.47%)
Mastitis, n (%)^b^	90 (6.74%)	16 (19.75%)^***^
Gestational week at delivery, mean (SD)^a^	39.091 (1.848)	38.527 (2.930)
Vaginal delivery, n (%)^b^	781 (58.46%)	49 (60.49%)
Epidural analgesia, n (%)^b^	584 (43.71%)	30 (37.04%)
Singleton, n (%)^b^	1315 (98.43%)	77 (95.06%)
First-time mothers, n (%)^b^	960 (71.86%)	54 (66.67%)^*^
Infant weight, g, mean (SD)^a^	3422.374 (714.933)	3425.987 (966.194)
Infant height, cm, mean (SD)^a^	50.261 (1.808)	50.208 (3.521)
Infant gender, male, n (%)^b^	657 (49.18%)	51 (53.09%)
Breast-feeding, n (%)^b^	1272 (95.21%)	70 (86.42%)^*^
Days from delivery to psychological assessment, d, mean (SD)^a^	52.317 (15.665)	51.588 (9.442)

Educational level, 1- no prior education, 2- Junior high school, 3- High school, 4- University and above

*HC* Healthy control, *PPD* Postpartum depression, *EPDS* Edinburgh Postnatal Depression Scale, *PHQ-9* Patient Health Questionnaire-9 scale, *SD* Standard deviation

^a^compared by Mann-Whitney *U* test, adjusted for multiple testing using the Benjamini-Hochberg procedure

^b^compared by the Chi-square test; p-values comparing PPD group with HC are indicated by asterisks as follows:

^*^p < 0.05

^**^p < 0.01

^***^p < 0.001

**Fig. 1:**
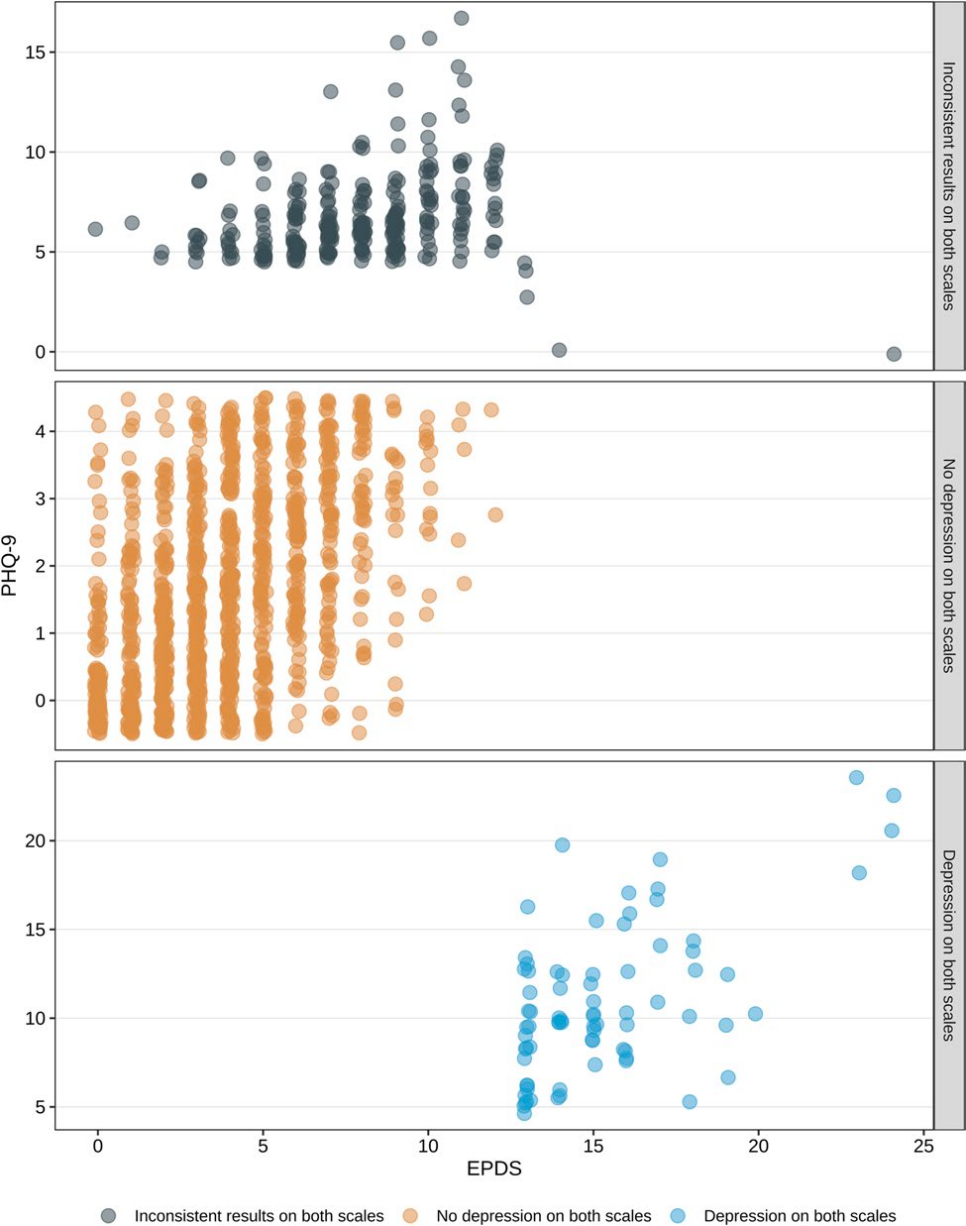
Comparison of the scores of the EPDS scale and the PHQ-9 scale for the same subject. EPDS, Edinburgh Postnatal Depression Scale; PHQ-9,the Patient Health Questionnaire-9 scale

PHQ-9 was performed to test the validity of EPDS. Both EPDS and PHQ-9 scores significantly differed among three groups and showed the same tendency. PHQ-9 average score was 10.370 in PPD group which was widely recognized as moderate to severe depression. However, when comparing the groups based on PHQ-9 and the groups based on EPDS, the results showed some inconsistency between EPDS and PHQ-9 (Fig. 1). Some women (12.85%) present contradictory results in EPDS and PHQ-9 included 156 women with normal EPDS scores but high PHQ-9 scores and 7 women with high EPDS scores but normal PHQ-9 scores.

### Attenuated and delayed niacin-induced skin flushing in PPD

**Fig. 2:**
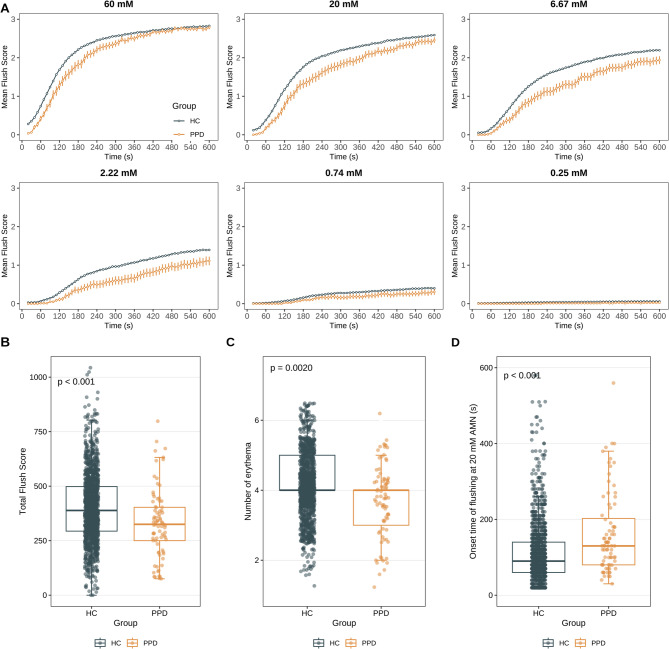
The distribution differences of nicotinic acid reaction between HC and PPD groups. **A** The time-flushing curves upon AMN stimulation of different concentrations. Flushing scores of the PPD group and HC group were shown with mean ± SEM. The horizontal axis represents reaction time, and the vertical axis represents average reaction score. **B**-**D** Boxplot with overlaid scatter points showing the distribution of total niacin-flushing scores **B**, number of erythema **C**, and onset time of flushing at 20 mM AMN **D** in the PPD group and HC group. The boxplot represents the median (central line), interquartile range (box boundaries), and 1.5× interquartile range (whiskers). Scatter points indicate individual data points, with jitter applied for better visualization. The p-value, compared by Mann-Whitney *U* test, adjusted for multiple testing using the Benjamini-Hochberg procedure. AMN, aqueous methyl nicotinate. SEM, standard error of the mean. PPD, postpartum depression. HC, healthy controls

The niacin-induced flushing response exhibited distinct characteristics between the PPD and HC groups. A line chart was used to illustrate the mean niacin-flushing scores (with SEM) for both the PPD and HC groups across various test concentrations and time points. The results demonstrated that, at each AMN concentration, the average niacin-flushing score in the PPD group was consistently lower than that in the HC group (Fig. 2A), with the between-group differences reaching statistical significance (Supplementary Table 1). Furthermore, the total niacin-flushing score in the PPD group was significantly reduced compared to the HC group (*p* < 0.001) (Fig. 2B). This disparity was also visually evident in the number of erythema patches observed on the participants’ arms at the conclusion of the test (*p* = 0.0020) (Fig. 2C). Specifically, the mean number of red patches in the PPD group was 3.728 ± 0.975 (mean ± SD), whereas the HC group exhibited a higher mean of 4.120 ± 0.850 (mean ± SD).

In addition to the variations in response degree, we also evaluated the response time of both groups to niacin stimulation, defined as the onset time of flushing for each participant. The PPD group consistently exhibited a slower response time compared to the HC group. Notably, under 20 mM concentration stimulation, the median response time for the PPD group was 130 s, whereas the HC group demonstrated a significantly faster median response time of 90 s (Fig. 2D). This disparity highlights that more than half of the HC group had developed two erythema patches within the first two minutes of testing, whereas the PPD group required more than two minutes for the second erythema to appear. Collectively, these findings demonstrate that both the intensity and temporal dynamics of the response are significantly diminished in the PPD group, indicating reduced sensitivity to niacin stimulation.

### The niacin blunted PPD women can be characterized by the flushing degree and the reaction rate

The reduced sensitivity of participants in the PPD group to nicotinic acid stimulation is primarily reflected in two key predictors: response time (the onset time of flushing at 20 mM AMN) and response intensity (the number of erythema). To evaluate the discriminatory potential of niacin-flushing in distinguishing between PPD and HC, the two key predictors were incorporated in the model. The ROC curves and AUC values for each fold was summarized in Supplementary Fig. 1. The mean AUC across all folds was 0.681 (95% CI: 0.588–0.775), indicating a potential ability of niacin- flushing to differentiate between PPD and HC groups. For postpartum women, the user-friendliness and practicality of the method take precedence over all other considerations. Therefore, we propose a stratified classification approach to differentiate between the PPD and HC groups. For postpartum women, the practicality and user-friendliness of screening methods are paramount. To address this, we developed a screening model using a two-step visual observation method, leveraging the distinct niacin-flushing response profiles observed in the PPD group.

**Fig. 3:**
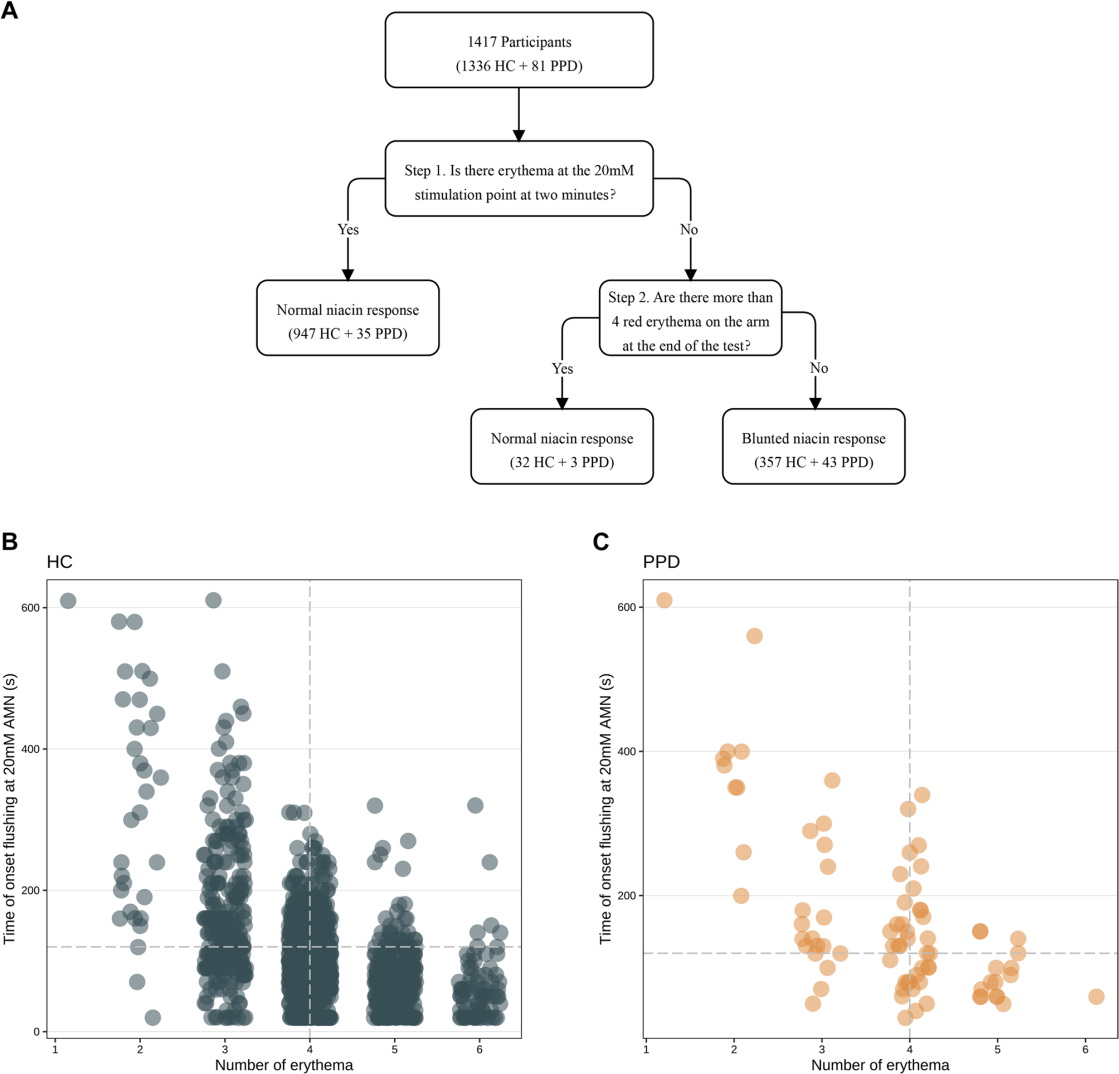
Identification of patients with PPD by a bivariate cut-off. **A** a two-step visual inspection method used to stratify the niacin-flushing response. **B** bivariate distribution of participants in PPD group and HC group. The vertical line indicates that the count of erythema at the end of the niacin test is 4. The horizontal line indicates that the flushing time under 20mM AMN stimulation is 130s. PPD, postpartum depression. HC, healthy controls

In the first step, we monitored the onset time of erythema induced by a 20 mM AMN concentration. Participants with a flushing time of less than 2 min were classified into the normal niacin-induced flushing subgroup, while those with a flushing time exceeding 2 min proceeded to the second step. In this step, we assessed the number of erythema patches on the participant’s arm at the conclusion of the niacin test. Individuals with more than 4 erythema patches were classified into the normal subgroup, whereas those with 4 or fewer were categorized as having diminished niacin flushing sensitivity (Fig. 3A). Using this two-step classification framework, the population distribution of PPD group and HC is shown in Fig. 3B. The screening model achieved a sensitivity of 53.09% (95% CI: 42.22% − 63.95%) and a specificity of 73.28% (95% CI: 70.90% − 75.65%) in distinguishing the PPD group from the HC group (Fig. 3B-C). The primary strength of this model lies in its exceptional user-friendliness, as the testing process relies on straightforward visual inspection that delivers immediate screening results.

### Niacin-flushing may indicate the prognosis of PPD

To further understanding the characteristics between the normal niacin-flushing response (NNR) group and blunted niacin-flushing response (BNR) group that were identified by the two-step visual observation method described above, we compared the subgroups with the clinical and demographic information (Table 2). Across the total cohort, PHQ-9 scores showed the same tendency in the subgroups, which women with moderate to severe depression symptom in PHQ-9 (69.77%) were mostly divided into the BNR subgroup in PPD group. Interestingly, results showed that BNR subgroup contained more breastfeeding mothers than NNR subgroup (93.02% vs. 86.84% in PPD group, *p* < 0.05, Fisher’s Exact test; 98.32% vs. 94.07 in HC group, *p* < 0.05, Fisher’s Exact test). This may be related to the increased consumption of PUFA by breast-feeding mothers. Moreover, a higher rate of lactation acute mastitis was in the BNR subgroup than the NNR subgroup (30.23% vs. 7.89% in PPD group, *p* < 0.05, Fisher’s Exact test; 8.96% vs. 5.92% in HC group), which suggests that mastitis may be a potential risk factor of PPD in breastfeeding women. Other clinical and demographic information was not found to be statistically significant difference in two subgroups. The data suggest that strengthening breastfeeding care in women may be one of the ways to prevent PPD.

**Table 2:** Characterization of a subgroup of women with a niacin-blunted phenotype

Items		PPD		HC	
		NNR	BNR	NNR	BNR
Total number, n		38	43	979	357
PHQ-9	Depression	42.10%	69.77%^a,*^	2.04%	1.12%
	Minor depression	39.47%	23.25%	17.57%	20.45%
	HC	5.26%	6.98%	79.79%	78.43%
Nursing way	Breast-feeding	86.84%	93.02% ^a,*^	94.07%	98.32%^b,*^
	Artificial breastfeeding	13.16%	6.98%	5.92%	1.68%
Mastitis	Yes	7.89%	30.23%	5.92%	8.96%
	No	92.11%	69.77%	94.08%	91.04%

*PPD* Postpartum depression, *HC* Healthy controls, *PHQ-9* Patient Health Questionnaire-9 scale

^a^comparison between BNR and NNR subgroups in PPD group

^b^comparison between BNR and NNR subgroups in HC group

^*^p < 0.05 by the Chi-square test or Fisher’s Exact test

## Discussions

### Principal findings

PPD populations exhibit attenuated and delayed niacin responses. Specifically, under the same stimulation conditions, compared with healthy people, the count of erythema in PPD people is less, and the time required for erythema to appear is longer. Based on these two characteristics, a screening standard for PPD was established, with a sensitivity of 53.09% (95% CI: 42.22% − 63.95%) and a specificity of 73.28% (95% CI: 70.90% − 75.65%).

### Results

In this study, we explored the screening potential of the niacin-blunted phenotype, an objective diagnostic marker of depression, in the PPD population. We clarified the characteristics of niacin skin flushing responses in a large cohort of pregnant women at 6 weeks postpartum. The results indicated that the PPD populations exhibited an attenuated and delayed niacin flushing response. The attenuation was reflected in a weaker degree of niacin-induced flushing and the delay was reflected in a slower rate of skin-flushing in the PPD group compared with the healthy mothers. Based on the two characteristics, a screening model of postpartum depression population was established. Under this criterion, women with PPD can be screened by observing the flushing status of subjects at two key time points during the niacin test. The first time point was whether the subject flushed when stimulated with 20 mM niacin for two minutes, representing the subject’s niacin flushing rate. The second time point was at ten minutes of niacin stimulation (the time point at the end of the test). The degree of niacin-flushing of the subjects was judged by observing the count of erythema on the subjects’ arms. Women with “no flushing at the first time point” and “less than five erythema at the second time point” were designated as niacin-blunted and at risk for PPD. The screening model had a sensitivity of 53.09% (95% CI: 42.22% − 63.95%) and a specificity of 73.28% (95% CI: 70.90% − 75.65%). The potential objective and use-friendly screening method provides a new idea for clinical diagnosis of PPD.

Interestingly, our study showed that the degree of the bluntness of niacin skin flushing response in PPD groups was weaker than that in depressed patients. It is reasonable that as a depressive state that occurs in a specific period, the clinical symptoms of PPD are not as severe as that of depression, and many patients with PPD can even recover on their own within 1 year after delivery. Furthermore, in this study, patients with PPD were screened based on the EPDS scale rather than clinician diagnosis, which also led to confounding in the results.

### Clinical implications

The niacin skin flushing response involves the niacin-AA-prostaglandin response pathway. Deficiency of AA in the epidermal cell membrane has been implicated as a major contributor to the niacin-blunted phenotype [20–25]. A growing body of research has provided compelling evidence suggesting that the niacin-flushing response may serve as a potential auxiliary diagnostic marker for a spectrum of major psychiatric disorders, including adult and adolescent depression, schizophrenia, bipolar disorder [10, 26–28]. The impaired niacin-flushing response in these psychiatric disorders is hypothesized to be associated with abnormal lipid metabolism and immune inflammation [29–31]. Coincidentally, studies on the pathological mechanisms of PPD have found that abnormal inflammation and lipid metabolism, were important risk factors for PPD [32, 33]. Changes in immune cells, such as fluctuations in monocyte counts, are believed to be associated with perinatal complications, including PPD and gestational hypertension [33, 34]. Additionally, abnormal levels of polyunsaturated fatty acids (PUFAs) have been widely reported in PPD. Specifically, patients with PPD had significantly lower levels of PUFAs than healthy mothers [35]. In addition, women with lower dietary n-3 PUFAs intake during pregnancy were at higher risk for PPD [36]. This suggests that the pathological mechanism of PPD shares a common molecular mechanism with the niacin-blunted phenotype.

In fact, mothers have been continuously depleting their own PUFA stores since pregnancy. Firstly, during pregnancy, the mother continuously and selectively supplies PUFAs in the body to the development of the fetal brain and nervous system through the placental transport system. Fetal brain development is primarily completed during pregnancy. The brain of a newborn is 75% the weight of an adult brain. Thus, maternal PUFAs are consumed in extremely large quantities during fetal maturation. During this process, the PUFAs with the highest transport rates were docosahexaenoic acid (DHA), AA and α-linolenic acid (ALA) [37]. In fact, the process of lipid metabolism is not only a source of nutrients for mother and baby, but also plays a role in the differentiation of fetal adipocytes. Abnormal lipid metabolism is a risk factor for the future development of metabolic disorders in mother and baby [38, 39]. Therefore, maintaining appropriate dietary outcomes during pregnancy to manage lipid metabolism plays a crucial role for both mother and fetus [38]. Secondly, during postpartum lactation, the mother continuously consumes PUFA in the body to maintain the nutritional level of breast milk. Kim et al. found that PUFA is one of the main components of breast milk, mainly AA and DHA [40]. Additionally, AA is an important regulatory mediator of prolactin. If the AA in the maternal body is excessively deprived, the prolactin cannot be properly regulated, which will eventually affect the secretion of milk [41, 42]. This is logically consistent with the research that breastfeeding can reduce the risk of PPD [43]. Therefore, although breastfeeding is encouraged to prevent PPD, attention should be paid to the maternal care to ensure the balance of PUFA in the diet and the nursing of the breast to prevent the occurrence of acute mastitis. It is conceivable that when the PUFA in the maternal body is excessively consumed but not replenished in time, the maternal health is threatened and PPD occurs. Therefore, we can reasonably speculate that the subgroup of PPD patients screened based on the niacin phenotype is precisely the patients with PPD caused by excessive PUFA deprivation. This will provide new ideas for the clinical intervention of PPD.

### Research implications

This study is a pioneering exploration of niacin phenotypic characteristics in a natural postpartum population. The clinical screening of PPD currently relies heavily on a series of depression screening scales. The lack of objectivity of existing methods brings great limitations to the prognosis and management of PPD. In the cohort recruited in this study, the subjects were screened for depressive mood by the EPDS and PHQ-9 scales, respectively. A considerable proportion of subjects showed inconsistency in the screening results of the two scales. The difference in the screening results, on the one hand, stemmed from the inconsistent evaluation standards for depression among different scales. On the other hand, subjects’ subjective concealment attitude, inaccurate understanding of questions due to their educational level, etc., would also affect the screening results. The exploration of objective diagnostic markers will hopefully break through the limitations of clinical screening for PPD.

### Strengths and limitations

Our study has several strengths. We established a screening technique for PPD based on niacin skin flushing response. This screening technique may be related to the excessive consumption of PUFA in maternal body, so it provides a new idea for objective screening and non-drug intervention of postpartum depression. What’s more, the screening technology is user-friendly and easy to promote in the postpartum depressed population.

However, it is important to acknowledge several limitations. Larger and more diverse outside cohorts are needed to confirm the robustness and reproducibility of the model across different populations. Future studies should aim to validate the screening model in multi-center settings with more heterogeneous samples to ensure its applicability in real-world clinical practice. Furthermore, the current model is exploratory in nature, and its performance metrics, while encouraging, should be interpreted with caution until further validation is conducted. Additionally, this study did not include prepregnancy data, which limits our ability to determine whether niacin-flushing changes are specific to the postpartum period or reflect pre-existing conditions. And the cross-sectional design lacks follow-up data, preventing us from assessing the stability and predictive value of niacin-flushing response over time. Meanwhile, potentially confounding variables such as socioeconomic variables and support networks that could influence both PPD and niacin-flushing were lacked and were not fully considered. The current study also excluded women who had any previous mental health diagnoses. While the exclusion criteria enhance internal validity for detecting PPD-specific signals, future research should examine niacin sensitivity in populations with psychiatric comorbidities to assess broader utility. And future studies should also incorporate prepregnancy data, longitudinal follow-up, and confounding variables to differentiate postpartum-specific changes from pre-existing conditions and to evaluate the long-term predictive value of niacin skin flushing response in postpartum depression.

## Conclusions

In the present study, we innovatively characterized the attenuated and delayed niacin flushing response in patients with PPD. Based on these characteristics, a potential PPD objective screening marker was established with a sensitivity of 53.09% and a specificity of 75.65%. However, the model remains to be optimized, which can be achieved by further recruiting clinically confirmed PPD patients. This study innovatively explores and provides new ideas for the screening and intervention of PPD.

## Supplementary Information

Supplementary Material 1.

Supplementary Material 2.

## Data Availability

The datasets used and/or analysed during the current study available from the corresponding authors (Professor Chunling Wan, Email: clwan@sjtu.edu.cn) on reasonable request.
