# Supplementary material for: Screening for individuals with postpartum depression by an attenuated and delayed niacin skin flushing response: a case control study
Source: BMC Pregnancy Childbirth. 2025 Sep 24;25:943. doi: 10.1186/s12884-025-08040-x (PMC12462174; doi:10.1186/s12884-025-08040-x)
Supplement: Supplementary file 2 — Supplementary Material 2. [file 12884_2025_8040_MOESM2_ESM.pdf]

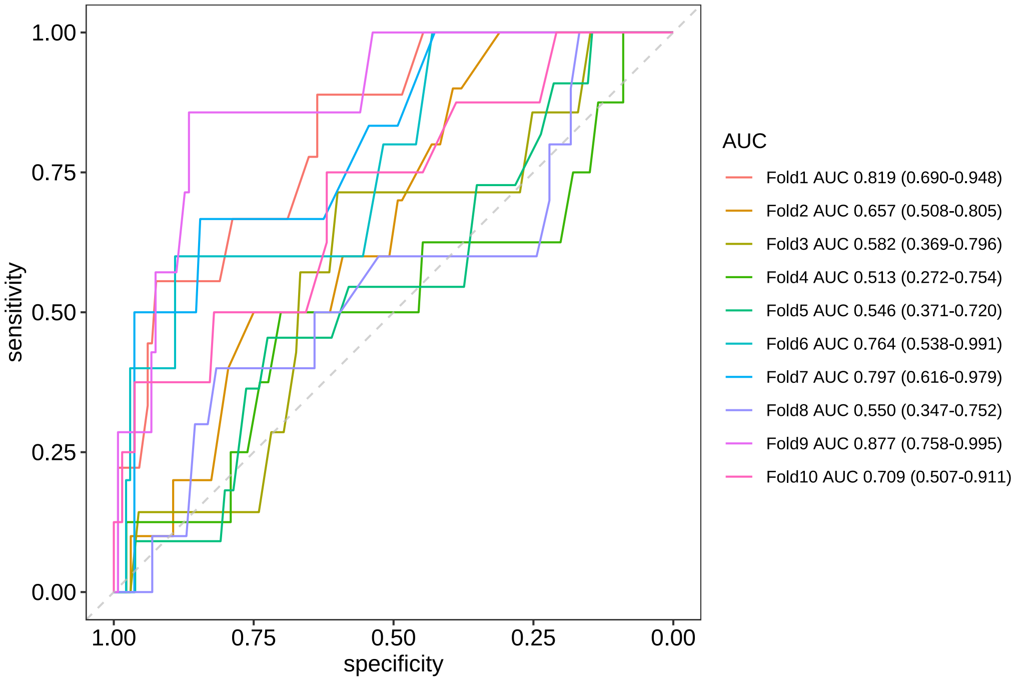


**Supplementary Figure 1. Receiver Operating Characteristic (ROC) curve for the binary logistic regression model using ten-fold cross-validation to assess the discriminative ability of the niacin-flushing phenotype between the PPD and HC groups.** The Area Under the Curve (AUC) with 95% confidence intervals (CIs) is computed using the DeLong method. Each fold represents the model's performance on the test set, demonstrating the predictive accuracy of the phenotype in distinguishing the two groups.
